# An analytical framework to derive the expected precision of genomic selection

**DOI:** 10.1186/s12711-017-0366-6

**Published:** 2017-12-27

**Authors:** Jean-Michel Elsen

**Affiliations:** GenPhySE (Génétique Physiologie et Systèmes d’Elevage), Université de Toulouse, INRA, ENVT, 31326 Castanet-Tolosan, France

## Abstract

**Background:**

Formulae to predict the precision or accuracy of genomic estimated breeding values (GEBV) are important when modelling selection schemes. Simple versions of such formulae have been proposed in the past, based on a number of simplifying hypotheses, including absence of linkage disequilibrium and linkage between loci, a population made up of unrelated individuals, and that all genetic variability of the trait is explained by the genotyped loci. These formulae were based on approximations that were not always clear. The objective of this paper is to offer a unique framework to derive equations that predict the precision of GEBV from the size of the reference population and the heritability of and number of QTL controlling the quantitative trait.

**Results:**

The exact formulation of the precision of GEBV involves the expectation of the inverse of a linear function of the genomic matrix, which cannot be calculated from simple algebra but can be approximated using a Taylor polynomial expansion. First order approximations performed better than the initial prediction equations published in the literature. Second order approximations produced almost perfect estimates of precision when compared to results obtained when simulating situations that agreed with the assumptions that were required to derive the precision equations. Using this proposed framework, we present several generalizations, including multi-trait genomic evaluation.

**Conclusions:**

Although further improvements are needed to account for the complexity of practical situations, the equations proposed here can be used to derive the precision of GEBV when comparing breeding schemes a priori.

**Electronic supplementary material:**

The online version of this article (10.1186/s12711-017-0366-6) contains supplementary material, which is available to authorized users.

## Background

After the seminal work of Meuwissen et al. [1], who provided statistical methods to exploit linkage disequilibrium (LD) between genotyped marker loci and quantitative trait loci (QTL) in animal and plant breeding, as previously proposed by Lande and Thompson [2], genomic selection was launched, which has since revolutionised both research in quantitative and applied genetics and practical breeding plans. The benefits of this technology are considerable in dairy cattle (e.g. [3–8]) and dairy cattle breeders very rapidly changed their schemes in order to adopt genomic selection methods. Thus, it became possible to improve the reliability of estimated breeding values (EBV) at a young age, avoid costly and lengthy progeny tests, and limit the detrimental evolution of inbreeding. However, the application of genomic selection was not so clear in other breeding sectors, for various reasons: the high relative costs of genotyping (compared to the value of reproducers), the limited size of the (reference) populations required to calibrate the effects of single nucleotide polymorphisms (SNPs), and the fact that basic schemes were already organised with short generation intervals (e.g. [9–13]).

Mathematical models to describe and evaluate breeding plans can be useful to decide whether a breeding scheme based on genomic evaluations should be implemented or not e.g. [14]. These models are often based on stochastic simulations, in which the characteristics of single individuals (their genotypes at a number of SNPs, including QTL located across the genome, and their phenotypes for traits influenced by QTL) are generated, in order to produce data files that can be used as in “real life” (e.g. [15, 16]). Alternatively, models that describe populations at a higher level (generations, cohorts, classes of reproducers defined by their role in the scheme) offer a more rapid and flexible alternative to evaluate alternate breeding programs. In such approaches, deterministic equations link population characteristics such as heritability, mean LD, replacement rates, and the number of genotyped individuals to expected genetic progress by unit of time. Some of the most important equations in these models are the formulae that predict the precision of genomic EBV (GEBV). Analyses of simulations and real data have clearly demonstrated that the precision of GEBV depends on the structure of the reference population and the characteristics of the marker set used. The size of this reference population, its diversity, the genetic distance between the reference population and the group of selection candidates genotyped, the number of markers, and the degree or strength of LD are the main factors that influence this precision [17–29].

A very simple formula to obtain the precision of GEBV was given by Daetwyler et al. [17], based on a number of simplifying hypotheses that included: absence of LD and linkage between loci, a population made up of unrelated individuals, and all genetic variation of the trait is explained by the genotyped loci. Under this approach, the regression of phenotypes on SNP genotypes was performed one locus at a time. This equation has been widely used and cited more than 100 times in the literature. Adjustments have since been proposed to deal with the distribution of marker allele frequencies [20], include dependence between marker loci through the definition of an effective number of independent loci [30], include the proportion of genetic variance explained by markers [22], and account for a smaller error variance when multiple marker loci are considered simultaneously [8]. Brard and Ricard [31] reviewed and challenged these formulae, using the results reported in 13 publications based on real data. They showed that the size of the reference population and the number of independent segments had a considerable impact on precision, and that the different formulae produced very different results. Other situations were explored by Hayes et al. [21] by considering dependence between the reference and candidate populations, by Wientjes et al. [32], who studied multi-population scenarios, and by Elsen [33], who suggested opportunities for the more systematic exploration of dependence between SNPs and between individuals.

In the present paper, using the simple situation that was initially studied by Daetwyler et al. [17], we propose a framework to derive equations that predict the precision of GEBV based on the size of the reference population, and the heritability of and number of QTL controlling the quantitative trait. We are interested in the expectation of the precision of GEBV, before implementing possible genotyping and selection schemes, as a tool for optimizing resources. With this prior approach, the variability summarized when computing the expectation of GEBV precision comes from marker locus polymorphisms as well as from QTL and environmental random effects. After demonstrating the performance of the solutions obtained, we explore extensions to more complex situations. Ten equations are successively proposed: (1) a general formulation of the expectation of the precision of GEBV; (2) the Daetwyler et al. [17] equation that assumes that the error variance is not modified after correction for SNP effects; (3) the Daetwyler et al. [17] equation that accounts for the corresponding reduction error variance; (4) an approximation of Eq. (1) based on a Taylor series expansion; (5) and (9) applications of Eq. (4) to the first order, assuming that all SNPs contribute equally to the genetic variance (Eq. 5) or that their effects share the same prior variance (Eq. 9); (6) an extension of Eq. (5) to the second order; (7) and (8) applications of Eq. (6) when assuming that distribution of allele frequencies is uniform (Eq. 7) or U-shaped (Eq. 8); (10) an extension to the multivariate situation.

## Methods

### Proposed framework

#### Notations and hypotheses

A list of abbreviations is in Table 1. Genomic predictions are based on a set of *M* biallelic SNPs, with alleles $A_{k}$ and *B*_*k*_ at locus *k*, the frequency of allele *B*_*k*_ being *f*_*k*_. All SNPs are assumed to be in linkage equilibrium. The reference population, which is considered to be a random subset of a larger population, is made up of *N* unrelated individuals, which are genotyped and phenotyped. We are interested in the precision of the GEBV of a selection candidate that is not related to individuals in the reference population, but belongs to a selection population that is another subset of the larger population. The GEBV is derived as a SNP best linear unbiased prediction (BLUP) based on SNP genotypes.

**Table 1:** List of abbreviations used in alphabetical order

Abbreviation	Full meaning
${\mathbf{a}}$	Vector of economic weights in $\gamma$
$\alpha$	Lower bound of the distribution of minor allele frequencies
$A_{k}$ and $B_{k}$	Alleles at SNP $k$
$\beta_{k}$	Effect of SNP $k$
$\hat{{\beta}}_{k}$	Prediction of the effect of SNP $k$
${\varvec{\upbeta}}_{j}$	Vector of SNP effects for trait $j$
${\varvec{\upbeta}}$	Vector of SNP effects
${\mathbf{B}}$	Covariance matrix $v\left( {\varvec{\upbeta}} \right)$
${\tilde{{\boldsymbol{\beta}}}}$	Estimates of fixed effects
$\gamma$	Selection objective
$\hat{\gamma }$	BLUP of $\gamma$
${\mathbf{D}}$	Expectation of ${\mathbf{X^{\prime}X}} + {\varvec{\Lambda}}$
$\delta_{k}$	$k^{th}$ diagonal term of ${\mathbf{D}}$
${\mathbf{E}}$	Deviation of ${\mathbf{X^{\prime}X}} + {\varvec{\Lambda}}$ from ${\mathbf{D}}$
${\mathbf{e}}$	Vector of residuals
$f_{min}$	Minimum minor allele frequency
$f_{k}$	Frequency of ${\text{allele }}B_{k}$
${\mathbf{F}}$	Matrix of SNP genotypes
$g$	Genetic value of the candidate
$\hat{g}$	GEBV of the candidate
${\mathbf{g}}$	Vector of genetic values
${\hat{\mathbf{g}}}$	BLUP of ${\mathbf{g}}$
$h_{0}^{2}$	Heritability
${\varvec{\Lambda}}$	Diagonal matrix with elements $\lambda_{k}$
$\lambda$	Ratio $\sigma_{e}^{2} /\sigma_{{{\boldsymbol{g}}_{k} }}^{2}$
$\lambda_{k}$	Ratio $\sigma_{e}^{2} /\sigma_{{{\boldsymbol{\beta}}_{k} }}^{2}$
$M_{e}$	Effective number of loci
$M$	Number of SNPs
$N_{e}$	Effective population size
$N$	Size of the reference population
${\mathbf{P}}$	Working matrix (${\mathbf{D}}^{ - 1} {\mathbf{ED}}^{ - 1} {\mathbf{E}}$)
${\mathbf{R}}$	Covariance matrix $v\left( {\mathbf{e}} \right)$
$\widetilde{{r^{2} }}$	Estimate of the precision of GEBV based on [17]
$\hat{r}^{2}$	Approximation of $r^{2}$ proposed here
$r^{2} \left( {{\mathbf{X}},{\mathbf{w}}} \right)$	Expected precision of GEBV, given $\varvec{X}$ and $\varvec{w}$
$r^{2}$	Marginal expected precision of GEBV
$\sigma_{{{\boldsymbol{\beta}}_{k} }}^{2}$	Variance of the effects $\beta_{k}$
$\sigma_{k}^{2}$	Variance of the number of $B_{k}$ alleles
$\sigma_{g}^{2}$	Genetic variance
$\sigma_{e}^{2}$	Environmental variance
$\sigma_{Y}^{2}$	Phenotypic variance
${\mathbf{w}}$	Vector of SNP genotypes
${\mathbf{X}}$	Genotype matrix
${\mathbf{y}}$	Vector of the phenotypes of the reference population

The random elements of the prediction model ${\mathbf{y}} = {\mathbf{X}} {\varvec{\upbeta}} + {\mathbf{e}}$ are as follows:

${\mathbf{y}}$ is a vector of phenotypes recorded in the reference population, assumed to be centred at zero. ${\varvec{\upbeta}}$ is a vector of SNP effects and is randomly distributed with a mean of 0 and covariance matrix $$v\left( {\varvec{\upbeta}} \right) = \left( {\begin{array}{*{20}c} {\sigma_{{{\boldsymbol{\beta}}_{1} }}^{2} } & \cdots & 0 \\ \vdots & \ddots & \vdots \\ 0 & \cdots & {\sigma_{{{\boldsymbol{\beta}}_{M} }}^{2} } \\ \end{array} } \right) = {\mathbf{B}}$$. Note that based on this matrix, the *β*_*k*_ effects are supposed to be uncorrelated.

${\mathbf{X}}$ is the genotype matrix defined by $X_{ik} = n_{ik} - 2f_{k}$, where *n*_*ik*_ ∊ {0, 1, 2} is the number of *B*_*k*_ alleles carried by individual *i* at locus *k*. We assume that allele frequencies *f*_*k*_ are known. The expectation of *X*_*ik*_ is null, and its variance is $\sigma_{k}^{2} = 2f_{k} \left( {1 - f_{k} } \right).$ Under linkage equilibrium between SNPs, the expectation of matrix ${\mathbf{X}}^{\prime}{\mathbf{X}}$ is $$N\left( {\begin{array}{*{20}c} {\sigma_{1}^{2} } & \cdots & 0 \\ \vdots & \ddots & \vdots \\ 0 & \cdots & {\sigma_{M}^{2} } \\ \end{array} } \right) = N{\mathbf{F}}$$.

All genetic variability is assumed to be explained by the SNPs.

${\mathbf{e}}$ is a vector of residuals with a mean of 0 and covariance matrix $$v\left( {\mathbf{e}} \right) = \left( {\begin{array}{*{20}c} {\sigma_{e}^{2} } & \cdots & 0 \\ \vdots & \ddots & \vdots \\ 0 & \cdots & {\sigma_{e}^{2} } \\ \end{array} } \right) = \sigma_{e}^{2} {\mathbf{I}}_{\varvec{N}} = {\mathbf{R}}$$.

$g = {{\bf w}}{\varvec{\upbeta}}$ is the true genomic breeding value of the candidate to be predicted, with ${\mathbf{w}}$ the vector of SNP genotypes, defined as the rows in ${\mathbf{X}}$. The variance of ${\mathbf{w}}$ is $v\left( {\mathbf{w}} \right) = {\mathbf{F}}$. Assuming all genetic variability is explained by the SNPs, we have $v\left( g \right) = E\left[ {{\mathbf{wBw^{\prime}}}} \right] = E\left[ {{\mathbf{XBX^{\prime}}}} \right]_{ii} \forall i$.

$\hat{g} = {\mathbf{w}} \hat{{\varvec{\upbeta}}}$ is the GEBV of the candidate, where ${\hat{{\varvec{\upbeta}}}} = ({\mathbf{X}}^{\prime}{\mathbf{R}}^{ - 1} {\mathbf{X}} + {\mathbf{B}}^{ - 1})^{ - 1} {\mathbf{X}}^{\prime}{\mathbf{R}}^{ - 1} {\mathbf{y}}$ is the BLUP of the SNP effects.

For a given set of genotypes ${\mathbf{X}}$, variance $v({\hat{\varvec{\upbeta}}}|{\mathbf{X}}) = {\mathbf{B}} - \left({\mathbf{X}}^{\prime}{\mathbf{R}}^{ - 1} {\mathbf{X}} + {\mathbf{B}}^{ - 1} \right)^{ - 1}$. Defining matrix $${\varvec{\Lambda}} = \left( {\begin{array}{*{20}c} {\lambda_{1} } & \cdots & 0 \\ \vdots & \ddots & \vdots \\ 0 & \cdots & {\lambda_{M} } \\ \end{array} } \right)$$, with $\lambda_{k} = \sigma_{e}^{2} /\sigma_{{{\boldsymbol{\beta}}_{k} }}^{2}$, this variance is also $v({{\hat{{\varvec{\upbeta}}}}}\left| {\mathbf{X}} \right.) = {\mathbf{B}} -\upsigma_{\text{e}}^{2} \left( {{\mathbf{X}}^{\prime}}{\mathbf{X}} + {\varvec{\Lambda}} \right)^{ - 1}$.

#### Expected precision of GEBV

Four sources of variation underlie the correlation between genomic breeding values (*g*) and their prediction ($\hat{g}$): the SNP genotypes (${\mathbf{X}}$ and ${\mathbf{w}}$), their effects (${\varvec{\upbeta}}$), and the environmental effects (${\mathbf{e}}$). Quite often, we are interested in the precision of GEBV, given the population genotypes (${\mathbf{X}}$ and ${\mathbf{w}}$), and the randomness arising from the variability of ${\varvec{\upbeta}}$ and ${\mathbf{e}}$, i.e. $r^{2} \left( {{\mathbf{X}},{\mathbf{w}}} \right) = \frac{{v\left( {\hat{g}|{\mathbf{X}},{\mathbf{w}}} \right)}}{{v\left( {g|{\mathbf{X}},{\mathbf{w}}} \right)}}$, which is a function of matrices ${\mathbf{X}}$ and ${\mathbf{w}}$. A priori, before genotyping, for instance when different SNP chip densities or reference population sizes are compared, the criterion of interest is $r^{2} = \frac{{v\left( {\hat{g}} \right)}}{v\left( g \right)}$. This is the situation explored in this paper.

The denominator in the previous equation for the precision of the GEBV is the genetic variance in the selection population: $v\left( g \right) = E_{w} \left[ {{\mathbf{wBw^{\prime}}}} \right] = tr\left[ {v\left[ {\mathbf{w}} \right]{\mathbf{B}}} \right] = tr\left[ {{\mathbf{FB}}} \right] = \mathop \sum \nolimits_{k} \sigma_{k}^{2} \sigma_{{{\boldsymbol{\beta}}_{k} }}^{2}.$ The variances of SNP effects are not known but must be estimated (e.g. [1]). In our a priori estimation of the precision of the GEBV, simplifying assumptions are needed. Following VanRaden [19], all variances of SNP effects are assumed to be equal to $\sigma_{\beta}^{2}$ and, thus, $\sigma_{g}^{2} = \sigma_{\beta}^{2}\sum \nolimits_{k}\sigma_{k}^{2}$. Alternatively, following Wientjes et al. [32], all SNPs contribute equally to $\sigma_{e}^{2} = \sigma_{g}^{2}$, i.e. $\sigma_{k}^{2} \sigma_{{{\boldsymbol{\beta}}_{k} }}^{2} = \sigma_{g}^{2} /M$. This is the situation considered in the present paper. The ratio *σ*_*e*_^2^/*σ*_*g*_^2^ will be denoted by *λ*.

The numerator of the equation for precision is $v\left( {\hat{g}} \right) = E_{X,w} \left[ {{\mathbf{w}}v\left( {{\hat{{\varvec{\upbeta}}}}|{\mathbf{X}},{\mathbf{w}}} \right){\mathbf{w^{\prime}}}} \right]$ since: (1) $v\left( {\hat{g}} \right) = E_{X,w} \left[ {v\left( {\hat{g}|{\mathbf{X}},{\mathbf{w}}} \right)} \right] + v_{X,w} \left[ {E\left( {\hat{g}|{\mathbf{X}},{\mathbf{w}}} \right)} \right]$ and (2) $E\left( {\hat{g}|{\mathbf{X}},{\mathbf{w}}} \right) = {\mathbf{w}}E\left( {{\hat{{\varvec{\upbeta}}}}|{\mathbf{X}}} \right) = 0$.

Since ${\hat{{\varvec{\upbeta}}}}$ and ${\mathbf{X}}$ are independent from ${\mathbf{w}} {\text{ and }}E\left[ {\mathbf{w}} \right] = 0$:$v\left( {\hat{g}} \right) = E_{w} \left[ {{\mathbf{w}}E_{X} \left[ {v\left( {{\hat{{\varvec{\upbeta}}}}|{\mathbf{X}}} \right)} \right]{\mathbf{w^{\prime}}}} \right] = tr\left[ {v\left[ {\mathbf{w}} \right]E_{X} \left[ {v\left( {{\hat{{\varvec{\upbeta}}}}|{\mathbf{X}}} \right)} \right]} \right]$.

Finally $$r^{2} = \frac{{ tr\left[ {{\mathbf{F}} {\mathbf{B}}} \right] - \sigma_{e}^{2} tr\left[ {{\mathbf{F}} E_{X} \left[ {\left( {{\mathbf{X}}^{\prime}{\mathbf{X}} + {\varvec{\Lambda}}} \right)^{ - 1} } \right]} \right]}}{{\sigma_{g}^{2} }}$$,

1 $${\text{i}} . {\text{e}} .\;r^{2} = 1 - \lambda \;tr\left[ {{\mathbf{F}}\;E_{X} \left[ {\left( {{\mathbf{X^{\prime}X}} + {\varvec{\Lambda}}} \right)^{ - 1} } \right]} \right].$$

#### Approximation of the precision of GEBV proposed by Daetwyler et al. [17]

In their derivation of the precision of GEBV, Daetwyler et al. [17] considered marker effects as both random and fixed effects. With our notations, they used ${\tilde{{\varvec{\upbeta}}}} = \left( {{\mathbf{X^{\prime}R}}^{ - 1} {\mathbf{X}}} \right)^{ - 1} {\mathbf{X}^{\prime}\mathbf{R}}^{ - 1} {\mathbf{y}} = \left( {{\mathbf{X}^{\prime}{\mathbf{X}}}} \right)^{ - 1} {\mathbf{X}}^{\prime}{\mathbf{y}}$ as a fixed effect estimator of ${\varvec{\upbeta}}$. In this context, $var\left( {{\tilde{{\varvec{\upbeta}}}} - {\varvec{\upbeta}}} \right) = var\left( {{\tilde{{\varvec{\upbeta}}}}} \right) = \left( {{\mathbf{X}^{\prime}\mathbf{X}}} \right)^{ - 1} \sigma_{e}^{2}$. However, when considering ${\varvec{\upbeta}}$ as a random effect, they used $cov\left( {{\varvec{\upbeta}},{\tilde{{\varvec{\upbeta}}}} - {\varvec{\upbeta}}} \right) = 0$, giving $cov\left( {\tilde{g},g} \right) = v\left( g \right),$ and found $r^{2} = \frac{ v\left( g \right) }{{v\left( {\tilde{g}} \right) }}$, i.e. the inverse of the classical $r^{2} = \frac{{ v\left( {\hat{g}} \right) }}{ v\left( g \right)}$. Assuming that SNPs are in linkage equilibrium, have uncorrelated effects, and independence between SNP effects and genotypes ($cov\left( {w_{j,} {{\upbeta}}_{j } } \right) = 0$), the variance $v\left( {\tilde{g}} \right) = v\left( {{\mathbf{w}}\left( {{\varvec{\upbeta}} + {\tilde{{\varvec{\upbeta}}}} - {\varvec{\upbeta}}} \right)} \right)$ is $\sum\nolimits_{j} {var\left( {w_{j } } \right)v\left( {\beta_{j} } \right)} + \sum\nolimits_{j} {var\left( {w_{j } } \right)v\left( {\tilde{\beta }_{j} - \beta_{j} } \right)}$. When the reference population size is sufficiently large, then $\left( {{\mathbf{X^{\prime}X}}} \right)^{ - 1} \approx E\left[ {\left( {{\mathbf{X^{\prime}X}}} \right)^{ - 1} } \right]$, giving $v\left( {\tilde{g}} \right) = \sigma_{g}^{2} + \sum\nolimits_{j} {{{var\left( {w_{j } } \right)\sigma_{e}^{2} } \mathord{\left/ {\vphantom {{var\left( {w_{j } } \right)\sigma_{e}^{2} } {Nvar\left( {x_{ij } } \right)}}} \right. \kern-0pt} {Nvar\left( {x_{ij } } \right)}}}$.

Initially, Daetwyler et al. [17] assumed inconsistently that both $\sigma_{p}^{2} = 1$ (“assuming the phenotypic variance is 1“) giving $\sigma_{g}^{2} = h_{0}^{1}$ and $\sigma_{e}^{2} = 1$ (“for the present, we shall conservatively take $\sigma_{e}^{2} = 1$”). Since the candidate and reference individuals belong to the same population, $var\left( {w_{j } } \right) = var\left( {x_{ij } } \right)$ and $v\left( {\tilde{g}} \right) = h_{0}^{2} + M/N$, which gives:

2 $$\widetilde{{r^{2} }}_{\left( 1 \right)} = \frac{{Nh_{0}^{2} }}{{Nh_{0}^{2} + M}}.$$

A correction was proposed to relax the approximation $\sigma_{e}^{2} = 1$, which resulted in an upward correction of $\widetilde{{r^{2} }}$. The idea was to replace $\sigma_{e}^{2} = 1$ by $\sigma_{e}^{2} = 1 - h_{0}^{2} + h_{0}^{2}(1 - r^{2})$, giving a quadratic equation in $r^{2}$ and

3 $$\widetilde{{r^{2} }}_{\left( 2 \right)} = \frac{{M + Nh_{0}^{2} \pm \sqrt {\left( {M + Nh_{0}^{2} } \right)^{2} - 4NMh_{0}^{4} } }}{{2Mh_{0}^{2} }}.$$

An alternative derivation of Eq. (2) was proposed by Wientjes et al. [32]. The main idea was that, assuming all SNPs are independent, their effects can be estimated in single random effect models, with ${\mathbf{y}} = {\mathbf{X}}_{k} \beta_{k} + {\mathbf{e}}_{k}$ for locus *k*, giving $\hat{\beta }_{k} = \left( {{\mathbf{X^{\prime}}}_{k} {\mathbf{X}}_{k} + \frac{{\sigma_{{e_{k} }}^{2} }}{{\sigma_{{\beta_{k} }}^{2} }}} \right)^{ - 1} {\mathbf{X^{\prime}}}_{k} {\mathbf{y}}$. They assumed that (1) the reference population was large (${\mathbf{X^{\prime}}}_{k} {\mathbf{X}}_{k} \approx N\sigma_{k}^{2}$), (2) the SNPs contributed equally to the genetic variance ($\sigma_{k}^{2} \sigma_{{\beta_{k} }}^{2} = \sigma_{g}^{2} /M$), and (3) the individual contribution of each SNP was very small ($\sigma_{{e_{k} }}^{2} = \sigma_{Y}^{2} - \sigma_{g}^{2} /M \cong \sigma_{Y}^{2}$). Applying these assumptions, the BLUP of *β*_*k*_ is $\hat{\beta }_{k} = \frac{{{\mathbf{X^{\prime}}}_{\varvec{k}} \varvec{y}}}{{\sigma_{k}^{2} \left( {N + M\sigma_{Y}^{2} /\sigma_{g}^{2} } \right)}}$, with variance $v\left( { \hat{\beta }_{k} } \right) = \frac{{N\sigma_{{\beta_{k} }}^{2} }}{{N + M/h_{0}^{2} }}$, and the precision of GEBV is $r^{2} = \frac{{v\left( {\hat{g}} \right)}}{v\left( g \right)} = \frac{{\varvec{w}v\left( {\hat{\varvec{\beta }}} \right)\varvec{w^{\prime}}}}{{\varvec{w}v\left(\varvec{\beta}\right)\varvec{w^{\prime}}}} = \frac{{\frac{N}{{N + M/h_{0}^{2} }}\mathop \sum \nolimits_{k} w_{k}^{2} \sigma_{{\beta_{k} }}^{2} }}{{\mathop \sum \nolimits_{k} w_{k}^{2} \sigma_{{\beta_{k} }}^{2} }} = \frac{{Nh_{0}^{2} }}{{Nh_{0}^{2} + M}}$.

#### Another approach to calculate the precision of GEBV

In the following, we do not assume that *σ*_*Y*_^2^ = *σ*_*e*_^2^ or $\sigma_{Y}^{2} = \sigma_{{e_{k} }}^{2}$, and a unique multi QTL random model (${\mathbf{y}} = {\mathbf{X}}{\varvec{\upbeta}} + {\mathbf{e}}$) is used to describe relationships between phenotype and genotype. As in Wientjes et al. [32], we assume that $\sigma_{k}^{2} \sigma_{{\beta_{k} }}^{2} = \sigma_{g}^{2} /M$. In Eq. (1), the expectation of the inverse of matrix ${\mathbf{X^{\prime}X}} + {\varvec{\Lambda}}$ appears. This matrix can be broken down into diagonal $\left( {{\mathbf{D}} = E\left[ {{\mathbf{X^{\prime}X}}} \right] + {\varvec{\Lambda}} } \right)$ and non-diagonal elements $\left( {{\mathbf{E}} = {\mathbf{X^{\prime}X}} - E\left[ {{\mathbf{X^{\prime}X}}} \right]} \right)$. As in Goddard et al. [22] and Elsen [33], a Taylor series expansion for matrix ${\mathbf{E}}$ is used to find approximations:

$$\begin{aligned}\left( {{\mathbf{X}}^{\prime}{\mathbf{X}} + {\varvec{\Lambda}}} \right)^{ - 1} &= \left( {{\mathbf{E}} + {\mathbf{D}}} \right)^{ - 1} = {\mathbf{D}}^{ - 1} \left( {{\mathbf{I}} + {\mathbf{ED}}^{ - 1} } \right)^{ - 1} \\ &= {\mathbf{D}}^{ - 1} \left( {{\mathbf{I}} - {\mathbf{ED}}^{ - 1} + {\mathbf{ED}}^{ - 1} {\mathbf{ED}}^{ - 1} \cdots } \right).\end{aligned}$$

Because ${\mathbf{D}}^{ - 1}$ is not random and $E_{X} \left[ {\mathbf{E}} \right] = 0$, the second order approximation is $E_{X} \left[ {\left( {{\mathbf{X^{\prime}X}} + {\varvec{\Lambda}}} \right)^{ - 1} } \right] = {\mathbf{D}}^{ - 1} + E_{X} \left[ {{\mathbf{D}}^{ - 1} {\mathbf{ED}}^{ - 1} {\mathbf{E}}} \right]{\mathbf{D}}^{ - 1}$ and the precision of GEBV can be approximated by:

4 $$\hat{r}^{2}_{\left( 2 \right)} \cong 1 - \lambda \left( {tr\left[ {\varvec{F} \varvec{D}^{ - 1} } \right] + tr\left[ {\varvec{F}E_{X} \left[ {\varvec{D}^{ - 1} \varvec{ED}^{ - 1} \varvec{E}} \right]\varvec{D}^{ - 1} } \right]} \right) .$$

#### First order approximation

Matrix ${\mathbf{D}}^{ - 1}$ is diagonal with terms $\frac{ 1}{{\delta_{k} }} = \frac{ 1}{{E\left[ {{\mathbf{X^{\prime}X}}} \right]_{kk} + \lambda_{k} }}$. In the first order, $\hat{r}^{2}_{\left( 1 \right)} \cong 1 - \frac{{ \sigma_{e}^{2} }}{{\sigma_{g}^{2} }}\left( {\mathop \sum \nolimits_{k} \frac{{ \sigma_{k}^{2} }}{{\delta_{k} }}} \right)$. Assuming as above that $\delta_{k} = N\sigma_{k}^{2} + \frac{{ \sigma_{e}^{2} }}{{\sigma_{{\beta_{k} }}^{2} }} = \sigma_{k}^{2} \left( {N + M\lambda } \right)$, we find:

5 $$\begin{aligned} &\hat{r}^{2}_{\left( 1 \right)} \cong 1 - \lambda \frac{M}{N + M\lambda }, \hfill \\ &\hat{r}^{2}_{\left( 1 \right)} = \frac{N }{N + M\lambda } = \frac{{Nh_{0}^{2} }}{{Nh_{0}^{2} + M\left( {1 - h_{0}^{2} } \right)}}. \hfill \\ \end{aligned}$$

This equation differs from formula (2) of Daetwyler et al. [17] by a factor (1 − *h*_0_^2^).

#### Second order approximation

When the size of the reference population is limited, elements of matrix ${\mathbf{X^{\prime}X}}$ differ from their expectations: non-zero non-diagonal terms are present even if the SNPs are in linkage equilibrium and diagonal elements diverge from the genetic variances. The second order approximation of Eq. (4) partly captures these deviations. In Eq. (4), matrices ${\mathbf{F}}$ and ${\mathbf{D}}^{ - 1}$ are both diagonal. Thus, we only need the diagonal of matrix ${\mathbf{P}} = {\mathbf{D}}^{ - 1} {\mathbf{ED}}^{ - 1} {\mathbf{E}}$ when computing the traces. Additional file 1 shows that terms *E*_*X*_[*P*_*kk*_] simplify to $\frac{{N\sigma_{k}^{2} }}{{\delta_{k} }}\left\{ {\frac{{1 - 2\sigma_{k}^{2} }}{{\delta_{k} }} + \mathop \sum \nolimits_{t} \frac{{\sigma_{t}^{2} }}{{\delta_{t} }}} \right\}$. A second order approximation of the precision of GEBV is thus $\hat{r}^{2}_{\left( 2 \right)} \cong 1 - \lambda \left( {\mathop \sum \nolimits_{k} \frac{{ \sigma_{k}^{2} }}{{\delta_{k} }}\left( {1 + \frac{{N\sigma_{k}^{2} }}{{\delta_{k} }}\left\{ {\frac{{1 - 2\sigma_{k}^{2} }}{{\delta_{k} }} + \mathop \sum \nolimits_{t} \frac{{\sigma_{t}^{2} }}{{\delta_{t} }}} \right\} } \right)} \right),$ which, using *δ*_*k*_ = *σ*_*k*_^2^(*N* + *Mλ*),  simplifies to:

6 $$\hat{r}^{2}_{\left( 2 \right)} \cong \frac{N }{N + M\lambda } - \lambda \frac{NM }{{\left( {N + M\lambda } \right)^{3} }} \left( {M - 2 + \frac{1}{M}\mathop \sum \limits_{k} \frac{ 1}{{\sigma_{k}^{2} }}} \right).$$

This expression includes genetic variances $\sigma_{k}^{2}$, which in practice can be estimated from the genomic data available. A priori, when these variances are not available, we can approximate the last term by using $E\left\lfloor {\frac{ 1}{{\sigma_{k}^{2} }}} \right\rfloor = E\left\lfloor\frac{ 1}{{2f_{k} \left( {1 - f_{k} } \right)}}\right\rfloor$. A general situation is a uniform distribution of the frequencies between *f*_*min*_, the minimum minor allele frequency of genotyped SNPs (MAF), and 1 − *f*_*min*_ (i.e. a probability density function $f\left( {f_{k} } \right) = \frac{1}{{1 - 2f_{min} }}$), which results in $E\left[\frac{ 1}{{\sigma_{k}^{2} }} \right]= \int_{{f_{min} }}^{{1 - f_{min} }} {\frac{1}{{1 - 2f_{min} }}} \frac{1}{2}\left[ {\frac{ 1}{{f_{k} }} + \frac{ 1}{{1 - f_{k} }}} \right]df_{k} = \frac{1}{{2\left( {1 - 2f_{min} } \right)}}\left[ {\log \left( {\frac{{1 - f_{min} }}{{f_{min} }}} \right) - \log \left( {\frac{{f_{min} }}{{1 - f_{min} }}} \right)} \right] = \frac{{\log \left( {\frac{{1 - f_{min} }}{{f_{min} }}} \right)}}{{1 - 2f_{min} }}$. A practical approximation of the expected precision of GEBV is thus:

7 $$\hat{r}^{2}_{\left( 2 \right)} \cong \frac{N }{N + M\lambda } - \lambda \frac{NM }{{\left( {N + M\lambda } \right)^{3} }} \left( {M - 2 + \frac{{\log \left( {\frac{{1 - f_{min} }}{{f_{min} }}} \right)}}{{1 - 2f_{min} }}} \right).$$

### Numerical comparison of estimates of the precision of GEBV

Equations (1), (2), (3), (5) and (7) were evaluated by simulating data corresponding to the hypotheses that underlie their development: unrelated reference and candidate individuals, SNPs in linkage equilibrium, GEBV from a SNP-based BLUP model, and all causal SNPs are included in the SNP panel. Heritability ranged from 0.1 to 0.7, genotypes were available for 500 or 1000 SNPs and the size of the reference population ranged from 1000 to 10,000. The minimum MAF (*f*_*min*_) was 0.025, 0.05, 0.075 or 0.10. One hundred replicates were simulated for each scenario, with allele frequencies generated for each. Computations were made in FORTRAN with the help of the Nag library [34].

**Fig. 1:**
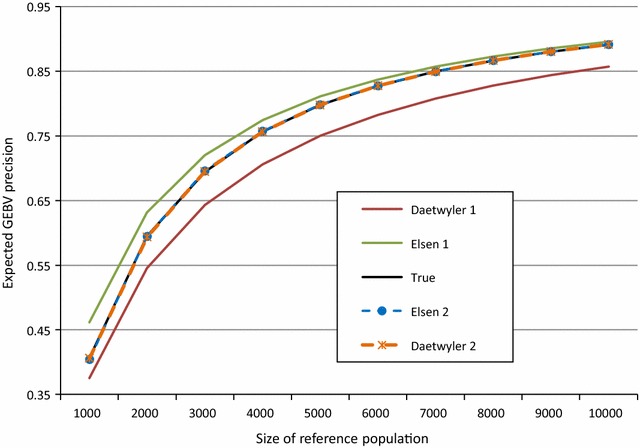
Expected precision of GEBV as predicted by Eqs. (1), (2), (3), (5) and (7), when 500 SNPs are genotyped, minimum minor allele frequency is 0.05, and heritability is 0.3

The results obtained when *M* = 500, *f*_*min*_ = 0.05 and *h*_0_^2^ = 0.3 are in Fig. 1. Table 2 shows the effects of heritability and *f*_*min*_ on the results. They showed consistently that (i) the prediction proposed by Daetwyler et al. [17] is closer to the “true” precision given by Eq. (1) when the size of the reference population is limited, (ii) the first order approximation proposed in the present paper (Eq. (5)) improves when the size of this population increases, i.e. when the assumptions that underlie these equations are more realistic, and (iii) the second order approximations of Eqs. (3) and (7) were nearly perfect in all cases, with a slight advantage for those of Daetwyler et al. [17] in most cases.

**Table 2:** Expected precision of GEBV as predicted by Eq. 1 (true precision), Daetwyler’s formula 2 and 3, Eqs. 5 and 7 when 500 SNPs are genotyped in a reference population of size 5000, depending on minimum minor allele frequency (MAF) and heritability

MAF	$\varvec{h}_{0}^{2}$	True Eq. (1)	Daetwyler formula (2)	Daetwyler formula (3)	Elsen Eq. (5)	Elsen Eq. (7)
0.05	0.1	0.514	0.500	0.513	0.526	0.513
0.05	0.3	0.798	0.750	0.798	0.811	0.798
0.05	0.5	0.901	0.833	0.901	0.909	0.902
0.05	0.7	0.955	0.875	0.955	0.959	0.955
0.025	0.1	0.513	0.500	0.513	0.526	0.513
0.025	0.3	0.797	0.750	0.798	0.811	0.798
0.025	0.5	0.901	0.833	0.901	0.909	0.902
0.025	0.7	0.955	0.875	0.955	0.959	0.955

### Extensions

The framework that we propose is flexible to accommodate alternative situations without major problems, as illustrated in the following.

#### Distribution of allelic frequencies

**Fig. 2:**
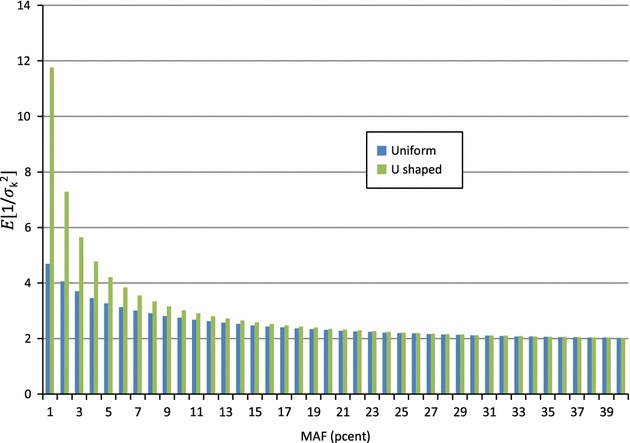
Expectation of the inverse of variance of allele frequencies as a function of minimum allele frequencies (MAF), assuming a uniform or U-shaped distribution of allele frequencies

In our a priori approach leading to Eq. (7), a uniform distribution of frequencies $f\left( {f_{k} } \right) = \frac{1}{{1 - 2f_{min} }}$ was assumed, corresponding to $E\left\lfloor {\frac{ 1}{{\sigma_{k}^{2} }}} \right\rfloor = \frac{{\log \left( {\frac{{1 - f_{min} }}{{f_{min} }}} \right)}}{{1 - 2f_{min} }}$. Following Hayes et al. [21], a U-shaped distribution could be assumed, with $f\left( {f_{k} } \right) = C/2f_{k} \left( {1 - f_{k} } \right)$. The *C* constant must be estimated from the constraint $\int_{\alpha }^{1 - \alpha } {f\left( {f_{k} } \right)df_{k} } = 1$, where *α* and 1 − *α* are the bounds of the *f*_*k*_ domain. Hayes et al. [21] argued that *α* = 1/2*N*_*e*_, with *N*_*e*_ being the effective size of the reference population, leading to *C* = 1/ log (2*N*_*e*_). Alternatively, we could set *α* = *f*_*min*_ and $E\left\lfloor {\frac{ 1}{{\sigma_{k}^{2} }}} \right\rfloor = 1 + \frac{{1 - 2f_{min} }}{{2f_{\text{min} } \left( {1 - f_{\text{min} } } \right)\log \left( {\frac{{1 - f_{min} }}{{f_{min} }}} \right)}}$. Figure 2 shows the values of these expectations for different minimum MAF. When the minimum MAF is higher than 5%, this correction factor $E\left\lfloor {\frac{ 1}{{\sigma_{k}^{2} }}} \right\rfloor$ is almost 2 and, in most cases, the expected precision of GEBV is close to:

8 $$\hat{r}^{2}_{\left( 2 \right)} \cong \frac{N }{N + M\lambda } - \lambda \left( {\frac{M }{N}} \right)^{2} \left( {\frac{N }{N + M\lambda }} \right)^{3} .$$

Goddard [20] also derived the precision of GEBV in this case of a U-shaped distribution of allele frequencies. In his formulation (formula (8) of his paper), the expectation of the precision of GEBV depends on the ratio $\lambda= \frac{{1 - h^{2} }}{{h^{2} }}\frac{{M_{e} }}{{\log \left( {2N_{e} } \right)}}$, where $M_{e}$ is the effective number of SNPs genotyped. Replacing log (2*N*_*e*_) by $- { \log }\left( {f_{min} } \right)$, as above, and assuming all SNPs are unlinked, resulting in *M*_*e*_ = *M*, we compared the two approaches by using simulated data, as explained in the previous section. Results in Table 3 suggest that, in most cases, Goddard’s formula underestimates the precision, while the two other formulations are close to the expected value obtained from simulation.

**Table 3:** Expected precision of GEBV when the distribution of allele frequencies is U-shaped, as predicted by Eqs. 1 and 8, by Daetwyler formula (3) and according to Goddard [20], when 500 SNPs are genotyped in a reference population of size 5000, depending on minimum minor allele frequency (MAF) and heritability

MAF	$\varvec{h}_{0}^{2}$	True Eq. (1)	Elsen Eq. (8)	Daetwyler formula (3)	Goddard [20]
0.05	0.1	0.513	0.513	0.513	0.491
0.05	0.3	0.798	0.798	0.798	0.759
0.05	0.5	0.901	0.902	0.901	0.867
0.05	0.7	0.955	0.955	0.955	0.930
0.025	0.1	0.513	0.513	0.513	0.537
0.025	0.3	0.798	0.798	0.798	0.791
0.025	0.5	0.901	0.901	0.901	0.886
0.025	0.7	0.955	0.955	0.955	0.941

#### Distribution of SNP effects

In previous developments, it was assumed that each SNP had its own distribution of effects with a variance of $\sigma_{{\beta_{k} }}^{2}$. This was the condition assumed by Meuwissen et al. [1] when defining BayesA and BayesB Markov chain Monte Carlo approaches to genomic evaluation. This was justified in practice because the authors did not work at a single locus level but considered haplotypes of markers around each tested position, while theoretical justifications were given in the Bayesian LASSO context by Park and Cassela [35]. Alternatively, Meuwissen et al. [1] considered a unique variance $\sigma_\beta^{2}$ under the GBLUP approach, which is also the case for the fitted SNPs in model BayesC $\uppi$ [36]. The assumption of an equal contribution of each SNP to the genetic variance is no longer valid and variance *σ*_*β*_^2^ is linked to genetic variance by $\sigma_{g}^{2} = \left( {\mathop \sum \nolimits_{k} \sigma_{k}^{2} } \right) \sigma_{\beta }^{2}$.

**Fig. 3:**
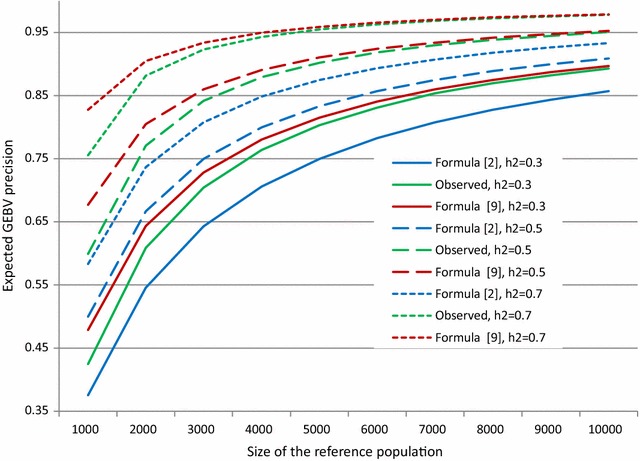
Expected precision of GEBV as predicted by Eqs. (1), (2) and (9) when 500 SNPs are genotyped and minimum minor allele frequency is 0.05. Case of a single prior variance of SNP effects and three levels of heritability (*h* ^2^ = 0.3, 0.5 and 0.7)

In this case, approximations of Eq. (1) for the expected precision of GEBV can be obtained using the same approach as before, using a matrix Taylor series expansion. As shown in Additional file 2, the first order approximation is given by:

9 $$\hat{r}^{2}_{\left( 9 \right)} = 1 - \frac{\lambda M}{N} \left( {1 + \frac{1}{{1 - 2 f_{min} }}\frac{{\hat{\lambda }_{\beta } }}{{N\sqrt {1 + \frac{{2\hat{\lambda }_{\beta } }}{N}} }}{ \log }\left( {\frac{{2 f_{min} - 1 + \sqrt {1 + \frac{{2\hat{\lambda }_{\beta } }}{N}} }}{{1 - 2 f_{min} + \sqrt {1 + \frac{{2\hat{\lambda }_{\beta } }}{N}} }}} \right)} \right),$$

where $\hat{\lambda }_{\beta } = \frac{M}{{1 - 2 f_{min} }}\frac{{1 - 6f_{min}^{2} + 2f_{min}^{3} }}{3}\lambda$. An illustration of the quality of this approximation is in Fig. 3. The quality of the first order approximation of Eq. (1), which always overestimates precision, increased with population size and heritability and appeared to be satisfactory when *N* ≥ 5000.

#### Multivariate prediction

A simple generalisation of the expected precision of GEBV can be obtained when retaining the previous assumption. A total of *n*_*c*_ traits are recorded in the reference population and this information is used to predict the global genetic value of the candidate: $\gamma = \sum\nolimits_{j} {a_{j} g_{j} } = {\mathbf{ag}}$, where ${\mathbf{a}}$ is a vector of *n*_*c*_ economic weights and ${\mathbf{g}}$ the column vector of *n*_*c*_ genetic values, i.e. $g_{j} = {\mathbf{w\beta }}_{j}$. Vector ${\varvec{\upbeta}}_{j}$ is a vector of the *M* SNP effects on trait *j* (${\varvec{\beta^{\prime}}}_{j} = \left( {\beta_{j1} , \ldots ,\beta_{jM} } \right)$). We assume that the vector of genotypes (${\mathbf{w}}$) is the same for all traits. All previous assumptions are retained: all SNPs have an effect on all traits, all SNPs have an equal contribution to genetic variance for each trait, and individuals are unrelated. SNP effects are distributed with specific prior variances of $\sigma_{{\beta_{jk} }}^{2}$, with zero correlations between SNPs. It is also assumed that the effects of SNP *k* on traits $j$ and $j^{\prime}$ are correlated, with a covariance of $\sigma_{{\beta_{jj'k} }}^{2}$.

The objective is to predict precision $r^{2} = \frac{{cov\left( {\gamma ,\hat{\gamma }} \right)^{2} }}{{v\left( \gamma \right)v\left( {\hat{\gamma }} \right)}}$, where $\hat{\gamma } = {\mathbf{a}}\hat{\mathbf{g}}$, with ${\hat{\mathbf{g}}}$ being the vector of GEBV. Thus, we need the variance $v\left( {{\hat{\mathbf{g}}}} \right)$ of these GEBV, a *n*_*c*_ × *n*_*c*_ matrix. As detailed in Additional file 3, this variance is estimated at the first order using:

10 $$\widehat{{v(\hat{\mathbf{g}})}} = Nv({\mathbf{g}})(Nv({\mathbf{g}}) + Mv({\mathbf{e}}))^{ - 1} v({\mathbf{g}}),$$

which is an obvious generalisation of the equivalent equation in the single-trait situation, which led to Eq. (5).

## Discussion

Using classical statistical theory, the expected precision of GEBV based on marker-based BLUP was derived simply. Numerical approximations, based on a matrix Taylor series expansion, were produced for simple situations. From simulations that were consistent with the assumptions corresponding to these situations, these approximations performed similarly to and often better than the formulae for precision of GEBV that were previously published. However, the framework developed here is simpler and enables direct generalisations.

The first order approximation proposed here (Eq. 5) differs from formula (2) of Daetwyler et al. [17] by a (1 − *h*_0_^2^) term. Those approximations differ by the way the error term variance is defined when a single SNP effect is estimated. In Eq. (2), it was assumed that this error term variance is the total phenotypic variance, because when estimating a unique SNP effect, all other SNP effects participate to the error term. Too much error is assumed with this approximation and the precision is under evaluated. Equation (5) behaves as if all other SNP effects were perfectly estimated, limiting the error term to the only non-genetic part. This gives an overestimation of the GEBV precision. The second order approximations try to correct for these under- or overestimations: Eq. (3) replaces the 1 − *h*_0_^2^ term of Eq. (5) by 1 − *r* ^2^ *h*_0_^2^, which corrects for the non-perfect estimation of other SNPs effects. Equation (7) accounts for the lack of orthogonality between the SNPs.

Asymptotic behaviours of first order approximations are not the same: when all the observed variability has an (additive) genetic origin, i.e. when *h*_0_^2^ = 1, formula (2) simplifies to $\frac{N }{N + M}$, while our Eq. (5) predicts a perfect precision of GEBV. This discrepancy disappears when correcting Eq. (2) for non-perfect estimation of other SNPS (Eq. 3). With this correction Eq. 3 predicts a perfect precision of GEBV when *h*_0_^2^ = 1. In spite of being algebraically very different, the second order approximations underlying the Eqs. (3) and (7) worked very similarly and produced results that were very close to those observed from simulations.

The hypotheses that underlie the equations derived here are strong and efforts should be made to overcome these constraints. First, it was assumed that all genetic variability is explained by the SNPs included in the evaluation. Although this is increasingly true as the size of SNP chips grows towards a full knowledge of genomes by resequencing and imputation, other polymorphisms, including copy number variations (CNV), may play a role and the genotype information obtained is still far from sufficient to fully explain genetic variability. It has been suggested [8, 22] that the proportion *b* of the genetic variance explained by the markers should be taken into account through a reduction in the heritability (from *h* ^2^ to *bh* ^2^) in the equations used and, using path coefficient theory, through a regression of precision of GEBV by *b* (from *r* ^2^ to *br* ^2^). This is easily implemented in the equations provided in this paper.

A second central hypothesis was independence between SNPs. With the current sizes of SNP chips, which will be even larger in the future, close SNPs are in LD and cannot be considered to be independent. This dependence means that non diagonal terms of $E\left[ {{\mathbf{X^{\prime}X}}} \right]$ are non-null, with $E\left[ {{\mathbf{X^{\prime}X}}} \right]_{kl} = 2N\Delta_{kl}$, where $\Delta_{kl}$ is the LD between SNPs *k *and *l*. Equations can be derived for this situation, based on principles similar to the theory given here, but they are cumbersome, e.g. [33]. The concept of effective independent chromosomal segments has been discussed [17] and formalised [20] as an alternative to the true number of markers. The idea is that the precision of the genomic prediction model “depends on the variation in the realised relationship between pairs of animals” [20]. Then, the effective number of loci is defined as the “number of independent loci that gives the same variance of realised relationships as obtained in the more realistic situation” [20]. Solutions have also been proposed to estimate the effective number of loci, from population genetics considerations [20, 21, 37] or from real data (e.g. [32]).

A third assumption was the absence of relationships between individuals in the reference population and between candidates in the reference population. A formalisation of situations where individuals are related was proposed [33] but only for the first order approximations of precision. Although relationships between reference individuals and candidates were accounted for by using this first order approximation, this was not the case for the structure of the reference population itself. Therefore, further efforts are needed, which is particularly important since it is clear that (1) genomic predictions of breeding values arise only partly from historical LD and increase in precision when individuals in the reference population and candidates are more closely related [26, 38–40], and (2) the structure of the reference population is a key factor in the precision on GEBV, e.g. [41].

The predicted variances of SNP effects calculated by Eq. (10) in the multivariate situation were obtained under strong assumptions. First, it was assumed that GEBV are computed using a multivariate approach that considers correlations between the effects of SNPs on different traits. However, in practice, GEBV are often computed using single-trait algorithms. In our formulation, this is equivalent to omitting the off-diagonal terms in matrices ${\mathbf{B}}_{kk}$ and ${\mathbf{E}}$ when estimating the SNP effects $\widehat{\underline{\boldsymbol{\upbeta}}}$. In this case, the variance of those effects, and $v\left( {{\hat{\mathbf{g}}}} \right)$, do not simplify to the equations derived in the case studied. A second important assumption was that all SNPs contributed equally to genetic covariances, as a direct extension of the single trait situation studied. The alternative assumption of unique (regardless of the SNPs) variances and covariances (e.g. $\sigma_{{\beta_{jk} }}^{2} = \sigma_{{\beta_{j} }}^{2} \forall k$) is also possible, as described in the previous section. Both these assumptions are, however, questionable, in particular because genetic correlations lower than 1 suggest that only a limited proportion of the SNPs (and underlying QTL) affect all traits. Extra prior information about the underlying genetic architecture of these correlations would be useful in this regard.

A few other assumptions are used in the current paper, including the additivity and *i.i.d.* of QTL effects, and the use of GBLUP. As long as the objective is to model and optimise breeding plans, then only relative values will be of interest and we assume that these assumptions are not critical for those comparisons.

## Conclusions

The objective of this paper was to provide a clear framework to derive predictive equations to estimate the precision of GEBV. Such equations can generate results in a second and thus enable the optimisation of a breeding program design through intensive numerical exploration. Not the entire complexity of practical breeding programs was included in the simple formulae derived here and in previously published papers. The purpose was to support the a priori comparison of breeding schemes, rather than to evaluate actual breeding schemes. The exact formulation of precision involves the expectation of the inverse of a linear function of the genomic relationship matrix, which cannot be calculated from simple algebra but can be approximated by a Taylor series development, as was already suggested by Goddard [20]. Second order approximations produced nearly perfect estimates of this precision, when compared to the results obtained by simulating data that are in agreement with the assumptions required to obtain the equations to estimate the precision of GEBV. We proposed several generalisations for the estimates of precision for this initial case, including multi-trait evaluation. Other situations can also be derived within the framework presented here.

## Additional files

**Additional file 1.** Diagonal elements of ${\text{E}}_{\text{X}} \left[ {{\text{D}}^{ - 1} {\text{ED}}^{ - 1} {\text{E}}} \right]$. This file provides details about the algebraic derivation of the expectation given in the title.

**Additional file 2.** Algebra when the SNP effect variance $\upsigma_{\upbeta}^{2}$ is unique. This file shows the derivation in the alternative hypothesis for the SNP effect variance.

**Additional file 3.** The multivariate case. This file provides details about the algebraic derivation in the multivariate case.
