# Supplementary material for: An analytical framework to derive the expected precision of genomic selection
Source: Genet Sel Evol. 2017 Dec 27;49:95. doi: 10.1186/s12711-017-0366-6 (PMC5745666; doi:10.1186/s12711-017-0366-6)
Supplement: Supplementary file 1 — Additional file 1. Diagonal elements of ${\text{E}}_{\text{X}} \left[ {{\text{D}}^{ - 1} {\text{ED}}^{ - 1} {\text{E}}} \right]$EXD-1ED-1E. This file provides details about the algebraic derivation of the expectation given in the title. [file 12711_2017_366_MOESM1_ESM.docx]

**Additional file 1: Diagonal elements of** $\boldsymbol{E}_{\boldsymbol{X}}\left[ \boldsymbol{D}^{\boldsymbol{-1}}\boldsymbol{E}\boldsymbol{D}^{\boldsymbol{-1}}\boldsymbol{E} \right]$

Elements of the matrix $\boldsymbol{P}=\boldsymbol{D}^{-1}\boldsymbol{E}\boldsymbol{D}^{-1}\boldsymbol{E}$ are $P_{kl}=\sum_{t} \frac{\left( \boldsymbol{X}^{\boldsymbol{'}}\boldsymbol{X}-E\left[ \boldsymbol{X}^{\boldsymbol{'}}\boldsymbol{X} \right] \right)_{kt}}{\delta_{k}}\frac{\left( \boldsymbol{X}^{\boldsymbol{'}}\boldsymbol{X}-E\left[ \boldsymbol{X}^{\boldsymbol{'}}\boldsymbol{X} \right] \right)_{tl}}{\delta_{t}}$

In the diagonal $P_{kk}=\sum_{t} \frac{\left( \boldsymbol{X}^{\boldsymbol{'}}\boldsymbol{X}-E\left[ \boldsymbol{X}^{\boldsymbol{'}}\boldsymbol{X} \right] \right)_{kt}}{\delta_{k}}\frac{\left( \boldsymbol{X}^{\boldsymbol{'}}\boldsymbol{X}-E\left[ \boldsymbol{X}^{\boldsymbol{'}}\boldsymbol{X} \right] \right)_{tk}}{\delta_{t}}=\sum_{t} \frac{\left( \sum_{i} x_{ik}x_{it}-{E\left[ X^{'}X \right]}_{kt} \right)^{2}}{\delta_{k}\delta_{t}}$

$P_{kk}=\frac{1}{\delta_{k}}\left\{ \frac{1}{\delta_{k}}\left( \sum_{i} x_{ik}^{2}-N\sigma_{k}^{2} \right)^{2}+\sum_{t\neq k} \frac{1}{\delta_{t}}\left( \sum_{i} x_{ik}x_{it} \right)^{2} \right\}$

The first term is $\left( \sum_{i} x_{ik}^{2}-N\sigma_{k}^{2} \right)^{2}=\left( \sum_{i} x_{ik}^{2} \right)^{2}-2N\sigma_{k}^{2}\left( \sum_{i} x_{ik}^{2} \right)+N^{2}\sigma_{k}^{4}$ with expectation

$E_{X}\left[ \left( \sum_{i} x_{ik}^{2}-N\sigma_{k}^{2} \right)^{2} \right]=\sum_{i} E_{X}\left[ x_{ik}^{4} \right]+\sum_{i} \sum_{j\neq i} E_{X}\left[ x_{ik}^{2} \right]E_{X}\left[ x_{jk}^{2} \right]-2N\sigma_{k}^{2}\left( \sum_{i} E_{X}\left[ x_{ik}^{2} \right] \right)+N^{2}\sigma_{k}^{4}$

It can simply be shown that$E_{X}\left[ x_{ik}^{4} \right]=E_{X}\left[ x_{ik}^{2} \right]=\sigma_{k}^{2}$, giving

$E_{X}\left[ \left( \sum_{i} x_{ik}^{2}-N\sigma_{k}^{2} \right)^{2} \right]=\sum_{i} \sigma_{k}^{2}+\sum_{i} \sum_{j\neq i} \sigma_{k}^{4}-2N\sigma_{k}^{2}\left( \sum_{i} \sigma_{k}^{2} \right)+N^{2}\sigma_{k}^{4}$ $=N\sigma_{k}^{2}-N\sigma_{k}^{4}$

Second term elements are such that$E_{X}\left[ \left( \sum_{i} x_{ik}x_{it} \right)^{2} \right]=\sum_{i} E_{X}\left[ \left( x_{ik}x_{it} \right)^{2} \right]+\sum_{i} \sum_{j\neq i} E_{X}\left[ x_{ik}x_{it}x_{jk}x_{jt} \right]$, with $E_{X}\left[ x_{ik}x_{it}x_{jk}x_{jt} \right]=0$ when $t\neq k$, leaving $E_{X}\left[ \left( \sum_{i} x_{ik}x_{it} \right)^{2} \right]=N\sigma_{k}^{2}\sigma_{t}^{2}$.

Then $E_{X}\left[ P_{kk} \right]=\frac{1}{\delta_{k}}\left\{ \frac{1}{\delta_{k}}N\sigma_{k}^{2}\left( 1-\sigma_{k}^{2} \right)+\sum_{t\neq k} \frac{N\sigma_{k}^{2}\sigma_{f_{t}}^{2}}{\delta_{t}} \right\}=\frac{N\sigma_{k}^{2}}{\delta_{k}}\left\{ \frac{1-2\sigma_{k}^{2}}{\delta_{k}}+\sum_{t} \frac{\sigma_{t}^{2}}{\delta_{t}} \right\}$
