# Supplementary material for: An analytical framework to derive the expected precision of genomic selection
Source: Genet Sel Evol. 2017 Dec 27;49:95. doi: 10.1186/s12711-017-0366-6 (PMC5745666; doi:10.1186/s12711-017-0366-6)
Supplement: Supplementary file 2 — Additional file 2. Algebra when the SNP effect variance $\upsigma_{\upbeta}^{2}$σβ2 is unique. This file shows the derivation in the alternative hypothesis for the SNP effect variance. [file 12711_2017_366_MOESM2_ESM.docx]

**Additional file 2: Algebra when the SNP effect variance** $\boldsymbol{\sigma}_{\boldsymbol{\beta}}^{\boldsymbol{2}}$ **is unique.**

Let $\lambda_{\beta}={\sigma_{e}^{2}}/{\sigma_{\beta}^{2}}$, with $\sigma_{\beta}^{2}\sum\sigma_{k}^{2}=\sigma_{g}^{2}$ The first order approximation of $\left[ 1 \right]$ is $\hat{r}^{2}=1-\lambda tr\left[ \boldsymbol{F} \boldsymbol{D}^{-1} \right]=1-\lambda\sum_{k} E\left[ \frac{\sigma_{k}^{2}}{N\sigma_{k}^{2}+\lambda_{\beta}} \right] .$The expectation $E\left[ \frac{\sigma_{k}^{2}}{N\sigma_{k}^{2}+\lambda_{\beta}} \right]=\frac{1}{N}E\left[ \frac{N\sigma_{k}^{2}+\lambda_{\beta}-\lambda_{\beta}}{N\sigma_{k}^{2}+\lambda_{\beta}} \right]=\frac{1}{N}\left( 1-E\left[ \frac{\lambda_{\beta}}{N\sigma_{k}^{2}+\lambda_{\beta}} \right] \right)=\frac{1}{N}\left( 1-\kappa E\left[ \frac{1}{f_{k}(1-f_{k})+\kappa} \right] \right)$with $\kappa=\frac{\lambda_{\beta}}{2N}$. Considering a uniform distribution of allele frequencies, we have $E\left[ \frac{1}{f_{k}(1-f_{k})+\kappa} \right]=-\frac{1}{\sqrt{1+4\kappa}}\frac{1}{1-2 f_{min}}\left( \int_{f_{min}}^{1-f_{min}} \frac{1}{f-\frac{1+\sqrt{1+4\kappa}}{2}}df-\int_{f_{min}}^{1-f_{min}} \frac{1}{f-\frac{1-\sqrt{1+4\kappa}}{2}}df \right)$

$E\left[ \frac{1}{f_{k}(1-f_{k})+\kappa} \right]=-\frac{1}{\sqrt{1+4\kappa}}{\frac{1}{1-2 f_{min}}\left[ log(\frac{\left| f-\frac{1+\sqrt{1+4\kappa}}{2} \right|}{\left| f-\frac{1-\sqrt{1+4\kappa}}{2} \right|}) \right]}_{f_{min}}^{1-f_{min}}$

As $1+4\kappa>1$, and $f_{min}>0$, $E\left[ \frac{1}{f_{k}(1-f_{k})+\kappa} \right]=-\frac{1}{\sqrt{1+4\kappa}}{\frac{1}{1-2 f_{min}}\left[ log(\frac{1+\sqrt{1+4\kappa}-2f}{2f-1+\sqrt{1+4\kappa}}) \right]}_{f_{min}}^{1-f_{min}}=-\frac{1}{1-2 f_{min}}\frac{2log(\frac{2 f_{min}-1+\sqrt{1+4\kappa}}{1-2 f_{min}+\sqrt{1+4\kappa}})}{\sqrt{1+4\alpha}}$

$$\hat{r}^{2}=1-\frac{\lambda M}{N} \left( 1+\frac{1}{1-2 f_{min}}\frac{\lambda_{\beta}}{N\sqrt{1+\frac{2\lambda_{\beta}}{N}}}\log\left( \frac{2 f_{min}-1+\sqrt{1+\frac{2\lambda_{\beta}}{N}}}{1-2 f_{min}+\sqrt{1+\frac{2\lambda_{\beta}}{N}}} \right) \right)$$

The coefficient $\lambda_{\beta}=\sum\sigma_{k}^{2}\lambda$ depends on the variances $\sigma_{k}^{2}$. With the same uniform hypothesis, its expectation is$\hat{\lambda}_{\beta}=\frac{M}{1-2 f_{min}}\frac{1-6{f_{min}}^{2}+2{f_{min}}^{3}}{3}\lambda$
