# Supplementary material for: An analytical framework to derive the expected precision of genomic selection
Source: Genet Sel Evol. 2017 Dec 27;49:95. doi: 10.1186/s12711-017-0366-6 (PMC5745666; doi:10.1186/s12711-017-0366-6)
Supplement: Supplementary file 3 — Additional file 3. The multivariate case. This file provides details about the algebraic derivation in the multivariate case. [file 12711_2017_366_MOESM3_ESM.docx]

**Additional file 3: The multivariate case.**

A simple generalisation of expected GEBV precision can be obtained while retaining the previous hypotheses. A total of $n_{c}$ traits are recorded in the reference population, and this information is used to predict the global genetic value of the candidate: $\gamma=\sum_{j} a_{j}g_{j}=\boldsymbol{ag}$ where $\boldsymbol{a}$is the line vector (dimension $n_{c}$) of economic weights and $\boldsymbol{g}$ the column vector (dimension $n_{c}$) of genetic values, *i.e.* the $g_{j}=\boldsymbol{w}\boldsymbol{\beta}_{\boldsymbol{j}}$. The term $\boldsymbol{\beta}_{\boldsymbol{j}}$ is the column vector of the $M$ SNP effects on trait $j$ ($\boldsymbol{\beta}_{\boldsymbol{j}}^{\boldsymbol{'}}=\left( \beta_{j1},\cdots,\beta_{jM} \right)$). Note that the genotype line vector ($\boldsymbol{w}$) is the same for all traits. As before, it will be assumed that all SNPs have an effect on the traits, and that these effects are distributed with specific prior variances $\sigma_{\beta_{jk}}^{2}$, without any correlations between SNPs. It will also be supposed that the effects of SNP $k$ on traits $j$and $j'$are correlated, with a covariance $\sigma_{\beta_{jj'k}}^{2}$.

The objective is to predict the precision $r^{2}=\frac{{cov(\gamma,\hat{\gamma})}^{2}}{v\left( \gamma\right)v(\hat{\gamma})}$, where$\hat{\gamma}=\boldsymbol{a}\hat{\boldsymbol{g}}$ with $\hat{\boldsymbol{g}}$ the GEBVs vector. We thus need the variance $v\left( \hat{\boldsymbol{g}} \right)$ of these genetic values, a $n_{c}\times n_{c}$ matrix.

Estimators ${\hat{\boldsymbol{\beta}}}_{\boldsymbol{j}}$ of SNP effects are obtained in the multitrait framework. Let the performance vector be sorted by individual and within individual by trait, and the SNP effects vector by SNP and within SNP by trait:

$${\underline{\boldsymbol{y}}}^{'}=\left( \left( y_{11},y_{12}\cdots y_{1n_{c}} \right),\cdots,\left( y_{N1},y_{N2}\cdots y_{Nn_{c}} \right) \right)=\left( \boldsymbol{y}_{\boldsymbol{1}}^{\boldsymbol{'}}\boldsymbol{,\cdots,}\boldsymbol{y}_{\boldsymbol{N}}^{\boldsymbol{'}} \right)$$

$${\underline{\boldsymbol{\beta}}}^{'}=\left( \left( \beta_{11},\beta_{21}\cdots\beta_{n_{c}1} \right),\cdots,\left( \beta_{1M},\beta_{2M}\cdots\beta_{n_{c}M} \right)+ \right)=\left( \boldsymbol{\beta}_{\boldsymbol{1}}^{\boldsymbol{'}}\boldsymbol{,\cdots,}\boldsymbol{\beta}_{\boldsymbol{M}}^{\boldsymbol{'}} \right)$$

$\left( \begin{matrix} \boldsymbol{y}_{\boldsymbol{1}} \\ \boldsymbol{y}_{\boldsymbol{i}} \\ \boldsymbol{y}_{\boldsymbol{N}} \end{matrix} \right)=\left( \begin{matrix} \boldsymbol{X}_{\boldsymbol{11}} & \boldsymbol{X}_{\boldsymbol{1}\boldsymbol{k}} & \boldsymbol{X}_{\boldsymbol{1}\boldsymbol{M}} \\ \boldsymbol{X}_{\boldsymbol{i}\boldsymbol{1}} & \boldsymbol{X}_{\boldsymbol{ik}} & \boldsymbol{X}_{\boldsymbol{iM}} \\ \boldsymbol{X}_{\boldsymbol{N}\boldsymbol{1}} & \boldsymbol{X}_{\boldsymbol{Nk}} & \boldsymbol{X}_{\boldsymbol{NM}} \end{matrix} \right)\left( \begin{matrix} \boldsymbol{\beta}_{\boldsymbol{1}} \\ \boldsymbol{\beta}_{\boldsymbol{k}} \\ \boldsymbol{\beta}_{\boldsymbol{M}} \end{matrix} \right)+\left( \begin{matrix} \boldsymbol{e}_{\boldsymbol{1}} \\ \boldsymbol{e}_{\boldsymbol{i}} \\ \boldsymbol{e}_{\boldsymbol{N}} \end{matrix} \right)$ or $\underline{\boldsymbol{y}}\boldsymbol{=}\underline{\boldsymbol{X}}\underline{\boldsymbol{\beta}}\boldsymbol{+}\underline{\boldsymbol{e}}$

The submatrices $\boldsymbol{X}_{\boldsymbol{ik}}$ are given by $\boldsymbol{X}_{\boldsymbol{ik}} =x_{ik}\boldsymbol{I}_{\boldsymbol{n}_{\boldsymbol{c}}}$ with $x_{ik}$ the k^th^ genotype of the i^th^ individual. The reference population genotype matrix is $\underline{\boldsymbol{X}}=\boldsymbol{X}\otimes\boldsymbol{I}_{\boldsymbol{n}_{\boldsymbol{c}}}$ with $\boldsymbol{X}$ the matrix for a single trait. The genotype matrix for the candidate genotype is $\underline{\boldsymbol{W}}=\boldsymbol{w}\otimes\boldsymbol{I}_{\boldsymbol{n}_{\boldsymbol{c}}}$, with $\boldsymbol{g}=\underline{\boldsymbol{W}}\underline{\boldsymbol{\beta}}$. Its variance $v\left( \underline{\boldsymbol{W}} \right)=\boldsymbol{F}\otimes\boldsymbol{I}_{\boldsymbol{n}_{\boldsymbol{c}}}$.

In this model, variances are such that$v\left( \underline{\boldsymbol{\beta}} \right)=\underline{\boldsymbol{B}}=\left( \begin{matrix} \boldsymbol{B}_{\boldsymbol{11}} & \boldsymbol{0} & \boldsymbol{0} \\ \boldsymbol{0} & \boldsymbol{B}_{\boldsymbol{kk}} & \boldsymbol{0} \\ \boldsymbol{0} & \boldsymbol{0} & \boldsymbol{B}_{\boldsymbol{MM}} \end{matrix} \right)$with $\boldsymbol{B}_{\boldsymbol{kk}}=\left( \begin{matrix} \sigma_{1k}^{2} & \sigma_{1jk} & \sigma_{1n_{c}k} \\ \sigma_{j1k} & \sigma_{jk}^{2} & \sigma_{jn_{c}k} \\ \sigma_{n_{c}1k} & \sigma_{n_{c}jk} & \sigma_{n_{c}k}^{2} \end{matrix} \right)$ and, retaining the hypothesis of an equal contribution of all SNP loci to genetic variance, the matrix $v\left( \boldsymbol{g}_{\boldsymbol{k}} \right)=\sigma_{k}^{2}\boldsymbol{B}_{\boldsymbol{kk}}=v(\boldsymbol{g})/M$

Still assuming independence between reference individuals,$\underline{\boldsymbol{R}}=\boldsymbol{I}_{\boldsymbol{N}}\otimes\boldsymbol{R}$ with $\boldsymbol{R}=\left( \begin{matrix} \sigma_{e1}^{2} & \sigma_{e1j} & \sigma_{e1n_{c}} \\ \sigma_{ej1} & \sigma_{ej}^{2} & \sigma_{e{jn}_{c}} \\ \sigma_{en_{c}1} & \sigma_{en_{c}j} & \sigma_{en_{c}}^{2} \end{matrix} \right)=v\left( \boldsymbol{e} \right)$.

All the developments given in the univariate case are still valid, in particular $\hat{\underline{\boldsymbol{\beta}}}=\left( {\underline{\boldsymbol{X}}}^{\boldsymbol{'}}{\underline{\boldsymbol{R}}}^{-1}\underline{\boldsymbol{X}}+{\underline{\boldsymbol{B}}}^{-1} \right)^{-1}\underline{\boldsymbol{X}}\boldsymbol{'}{\underline{\boldsymbol{R}}}^{\boldsymbol{-1}}\underline{\boldsymbol{y}}$ and $v\left( \underline{\hat{\boldsymbol{\beta}}}\boldsymbol{|}\underline{\boldsymbol{X}} \right)= \underline{\boldsymbol{B}}-\left( {\underline{\boldsymbol{X}}}^{\boldsymbol{'}}{\underline{\boldsymbol{R}}}^{-1}\underline{\boldsymbol{X}}+{\underline{\boldsymbol{B}}}^{-1} \right)^{-1}$. Using basics with respect to Kronecker products, we have ${\underline{\boldsymbol{X}}}^{\boldsymbol{'}}{\underline{\boldsymbol{R}}}^{-1}\underline{\boldsymbol{X}}\boldsymbol{=} \left( \boldsymbol{X}\otimes\boldsymbol{I}_{\boldsymbol{n}_{\boldsymbol{c}}} \right)^{'}\left( \boldsymbol{I}_{\boldsymbol{N}}\otimes\boldsymbol{R} \right)^{-1}\left( \boldsymbol{X}\otimes\boldsymbol{I}_{\boldsymbol{n}_{\boldsymbol{c}}} \right)=\left( \boldsymbol{X'}\otimes\boldsymbol{I}_{\boldsymbol{n}_{\boldsymbol{c}}} \right)\left( \boldsymbol{I}_{\boldsymbol{N}}\otimes\boldsymbol{R}^{-1} \right)\left( \boldsymbol{X}\otimes\boldsymbol{I}_{\boldsymbol{n}_{\boldsymbol{c}}} \right)=\left( \boldsymbol{X'}\otimes\boldsymbol{I}_{\boldsymbol{n}_{\boldsymbol{c}}} \right)\left( \boldsymbol{I}_{\boldsymbol{N}}\boldsymbol{X}\otimes\boldsymbol{R}^{-1}\boldsymbol{I}_{\boldsymbol{n}_{\boldsymbol{c}}} \right)=\boldsymbol{X'}\boldsymbol{I}_{\boldsymbol{N}}\boldsymbol{X}\otimes\boldsymbol{I}_{\boldsymbol{n}_{\boldsymbol{c}}}\boldsymbol{R}^{-1}\boldsymbol{I}_{\boldsymbol{n}_{\boldsymbol{c}}}=\boldsymbol{X'X}\otimes\boldsymbol{R}^{-1}$.

Then ${\underline{\boldsymbol{X}}}^{\boldsymbol{'}}{\underline{\boldsymbol{R}}}^{-1}\underline{\boldsymbol{X}}+{\underline{\boldsymbol{B}}}^{-1}\boldsymbol{=} \left[ \left( \boldsymbol{X'X}\otimes\boldsymbol{R}^{-1} \right)\left( \boldsymbol{I}_{\boldsymbol{M}}\otimes\boldsymbol{R} \right)+{\underline{\boldsymbol{B}}}^{-1}\left( \boldsymbol{I}_{\boldsymbol{M}}\otimes\boldsymbol{R} \right) \right]\left( \boldsymbol{I}_{\boldsymbol{M}}\otimes\boldsymbol{R} \right)^{-1}$

*i.e.* $\left[ \left( \boldsymbol{X'X}\otimes\boldsymbol{I}_{\boldsymbol{n}_{\boldsymbol{c}}} \right)+{\underline{\boldsymbol{B}}}^{-1}\left( \boldsymbol{I}_{\boldsymbol{M}}\otimes\boldsymbol{R} \right) \right]\left( \boldsymbol{I}_{\boldsymbol{M}}\otimes\boldsymbol{R}^{-1} \right)$, giving $v\left( \hat{\underline{\boldsymbol{\beta}}}\boldsymbol{|}\underline{\boldsymbol{X}} \right)= \underline{\boldsymbol{B}}-\left( \boldsymbol{I}_{\boldsymbol{M}}\otimes\boldsymbol{R} \right)\left( \left( \boldsymbol{X'X}\otimes\boldsymbol{I}_{\boldsymbol{n}_{\boldsymbol{c}}} \right)+{\underline{\boldsymbol{B}}}^{-1}\left( \boldsymbol{I}_{\boldsymbol{M}}\otimes\boldsymbol{R} \right) \right)^{-1}$. The first term of the inverse matrix comprises $M\times M$ diagonal blocks with terms $\left( \left[ \boldsymbol{X}^{\boldsymbol{'}}\boldsymbol{X} \right]_{kl} \right)\boldsymbol{I}_{\boldsymbol{n}_{\boldsymbol{c}}}$ while the second term is block diagonal with $M$ blocks $\boldsymbol{B}_{\boldsymbol{kk}}^{-1}\boldsymbol{R}$ .

Using algebra similar to the univariate case, and the fact that $E\left( \hat{\underline{\boldsymbol{\beta}}} \right)=E\left( \hat{\underline{\boldsymbol{\beta}}}\boldsymbol{|}\underline{\boldsymbol{X}} \right)=\boldsymbol{0},$the genetic variance matrix $v\left( \hat{\boldsymbol{g}} \right)$ is given by

$v\left( \hat{\boldsymbol{g}} \right)=v\left( \underline{\boldsymbol{W}}\hat{\underline{\boldsymbol{\beta}}} \right)=E_{\boldsymbol{W}}\left[ \underline{\boldsymbol{W}}E_{\boldsymbol{X}}\left( v\left( \underline{\hat{\boldsymbol{\beta}}}\boldsymbol{|}\underline{\boldsymbol{X}} \right) \right)\underline{\boldsymbol{W}}\boldsymbol{'} \right]=v\left( \boldsymbol{g} \right)-E_{\boldsymbol{W}}\left[ \underline{\boldsymbol{W}}E_{\boldsymbol{X}}\left( \left( \boldsymbol{I}_{\boldsymbol{M}}\otimes\boldsymbol{R} \right)\left( \left( \boldsymbol{X'X}\otimes\boldsymbol{I}_{\boldsymbol{n}_{\boldsymbol{c}}} \right)+{\underline{\boldsymbol{B}}}^{-1}\left( \boldsymbol{I}_{\boldsymbol{M}}\otimes\boldsymbol{R} \right) \right)^{-1} \right)\underline{\boldsymbol{W}}\boldsymbol{'} \right]$

Still using the decomposition ${\underline{\boldsymbol{X}}}^{\boldsymbol{'}}\underline{\boldsymbol{X}}=E\left[ {\underline{\boldsymbol{X}}}^{\boldsymbol{'}}\underline{\boldsymbol{X}} \right]+{\underline{\boldsymbol{X}}}^{\boldsymbol{'}}\underline{\boldsymbol{X}}-E\left[ {\underline{\boldsymbol{X}}}^{\boldsymbol{'}}\underline{\boldsymbol{X}} \right]$ and a Taylor development, the first order approximation of the variance matrix is

$$\hat{v\left( \hat{\boldsymbol{g}} \right)}=v\left( \boldsymbol{g} \right)-E_{W}\left[ \underline{\boldsymbol{W}}\left( \boldsymbol{I}_{\boldsymbol{M}}\otimes\boldsymbol{R} \right)\left( \left( E\left[ {\underline{\boldsymbol{X}}}^{\boldsymbol{'}}\underline{\boldsymbol{X}} \right]\otimes\boldsymbol{I}_{\boldsymbol{n}_{\boldsymbol{c}}} \right)+{\underline{\boldsymbol{B}}}^{-1}\left( \boldsymbol{I}_{\boldsymbol{M}}\otimes\boldsymbol{R} \right) \right)^{-1}\underline{\boldsymbol{W}}\boldsymbol{'} \right]$$

Here the inverse matrix is bloc diagonal, with $M$ blocs of dimensions $n_{c}{\times n}_{c} : N\sigma_{k}^{2}\boldsymbol{I}_{\boldsymbol{n}_{\boldsymbol{c}}}+\boldsymbol{B}_{\boldsymbol{kk}}^{-1}\boldsymbol{R}$. Thus the term under expectation is also bloc diagonal with terms $\boldsymbol{R}\left( N\sigma_{k}^{2}\boldsymbol{B}_{\boldsymbol{kk}}+\boldsymbol{R} \right)^{-1}\boldsymbol{B}_{\boldsymbol{kk}}$, and finally

$$\hat{v\left( \hat{\boldsymbol{g}} \right)}=v\left( \boldsymbol{g} \right)-E_{W}\left[ \sum_{k} w_{k}\boldsymbol{R}\left( N\sigma_{k}^{2}\boldsymbol{B}_{\boldsymbol{kk}}+\boldsymbol{R} \right)^{-1}{{\boldsymbol{B}_{\boldsymbol{kk}}\boldsymbol{I}}_{\boldsymbol{n}_{\boldsymbol{c}}}w}_{k} \right]$$

$$\hat{v\left( \hat{\boldsymbol{g}} \right)}=v\left( \boldsymbol{g} \right)-\boldsymbol{R}\sum_{k} \left( N\sigma_{k}^{2}\boldsymbol{B}_{\boldsymbol{kk}}+\boldsymbol{R} \right)^{-1}{\sigma_{k}^{2}\boldsymbol{B}}_{\boldsymbol{kk}}=v\left( \boldsymbol{g} \right)-\boldsymbol{R}\left( Nv\left( \boldsymbol{g} \right)/M+\boldsymbol{R} \right)^{-1}v\left( \boldsymbol{g} \right)$$

$$\hat{v\left( \hat{\boldsymbol{g}} \right)}=v\left( \boldsymbol{g} \right)-Mv\left( \boldsymbol{e} \right)\left( Nv\left( \boldsymbol{g} \right)+Mv\left( \boldsymbol{e} \right) \right)^{-1}v\left( \boldsymbol{g} \right)$$

$$\hat{v\left( \hat{\boldsymbol{g}} \right)}=Nv\left( \boldsymbol{g} \right)\left( Nv\left( \boldsymbol{g} \right)+Mv\left( \boldsymbol{e} \right) \right)^{-1}v\left( \boldsymbol{g} \right)$$
